# Methylenetetrahydrofolate reductase polymorphism 677C>T is associated with peripheral arterial disease in type 2 diabetes

**DOI:** 10.1186/1475-2840-4-17

**Published:** 2005-11-07

**Authors:** Rebecca L Pollex, Mary Mamakeesick, Bernard Zinman, Stewart B Harris, Anthony JG Hanley, Robert A Hegele

**Affiliations:** 1 Robarts Research Institute, London, Ontario, Canada; 2 Sandy Lake Health and Diabetes Project, Sandy Lake, Ontario, Canada; 3 Department of Medicine, University of Toronto and Leadership Sinai Centre for Diabetes, Mount Sinai Hospital, Toronto, Ontario, Canada; 4 Thames Valley Family Practice Research Unit, University of Western Ontario, London, Ontario, Canada

## Abstract

**Background:**

Individuals with diabetes are twice as likely to develop peripheral arterial disease (PAD), the manifestation of extensive atherosclerosis throughout the lower extremities. One putative determinant of PAD is the 677C>T polymorphism in the gene encoding methylenetetrahydrofolate reductase (*MTHFR*), which has previously been found to associate with various diabetic complications including retinopathy, nephropathy, atherosclerosis and coronary heart disease. The objective of this study was to investigate a possible role for the MTHFR 677C>T gene polymorphism with PAD in subjects with type 2 diabetes from an isolated aboriginal Canadian population.

**Methods:**

The 677C>T MTHFR gene polymorphism was genotyped in 138 subjects of Oji-Cree descent. Participants were selected from a community-wide survey that included PAD assessment by ankle-brachial index (ABI) measurement, and also intermittent claudication assessment by the Rose questionnaire.

**Results:**

*MTHFR *677T allele carriers had an increased risk of PAD with an odds ratio of 3.54 (95% CI 1.01, 12.4), *P *= 0.049, after adjustment for age, sex, duration of diabetes, hypertension, current smoking habits, and use of insulin or oral treatment for diabetes. None of these additional co-variables was significantly associated with PAD. No association was found between *MTHFR *genotype and intermittent claudication.

**Conclusion:**

The genetic influence of the *MTHFR *677C>T genotype on diabetic PAD is modest, yet for the Oji-Cree it is a major risk factor in comparison to other traditional risk factors.

## Background

Peripheral arterial disease (PAD) of the lower extremities, due to atherosclerosis occurring at the aortic bifurcation, femoral and popliteal arteries, commonly manifests as asymptomatic changes in intermediate phenotypes such as the ankle-brachial index (ABI). The most common symptom experienced is the aches and pains of intermittent claudication, and in extreme situations, individuals may develop gangrene and require lower-limb amputations [1]. While most subjects with PAD remain asymptomatic, all have markedly increased risk of developing cardiovascular disease, as PAD is fundamentally a sign of systemic atherosclerosis. Compared with age-matched controls, patients with intermittent claudication have a threefold increase in cardiovascular mortality [2,3] and a low ABI has been shown to be an independent predictor of both all-cause and cardiovascular mortality [4,5]. The presence of diabetes is associated with a doubling of PAD risk [6]; up to 15% of subjects with type 2 diabetes can have clinically significant PAD [6,7]. Other well-known risk factors for PAD include hypertension, dyslipidemia, and smoking [1].

Efforts to determine some of the genetic factors underlying susceptibility to PAD have met limited success [8-14]. Candidate genes include those that have shown association with atherosclerosis in other vascular beds. One such candidate is the gene encoding methylenetetrahydrofolate reductase (MTHFR), a key enzyme in the alternative pathway of homocysteine metabolism that remethylates homocysteine to methionine. Elevated plasma homocysteine has been reported among carriers of the *MTHFR *677C>T (Ala222Val; MIM 607093.0003) thermolabile single nucleotide polymorphism (SNP) [15]. This dysfunctional SNP is associated with reduced enzyme activity, resulting in a relative deficiency in the remethylation process [15], leading to elevated plasma homocysteine. Elevated plasma homocysteine concentration appears to be significantly associated with PAD [9,16]. Hyperhomocysteinemia may promote vascular disease through endothelial injury, predisposing the vessel to atherosclerosis [17]. Since the *MTHFR *677C>T SNP is an important determinant of plasma homocysteine concentration, this polymorphism may represent an important genetic risk factor in vascular disease.

The *MTHFR *677C>T SNP has previously been found to associate with various diabetic complications, including retinopathy [18-21], nephropathy [22-25], atherosclerosis [26], and coronary heart disease [27]. However, no studies to date have reported an association specifically for PAD, as measured, for instance, by the non-invasive ABI, a tool that has proven its usefulness in predicting future cardiovascular events [28]. Thus the objective of this study was to investigate a possible role for the *MTHFR *677C>T polymorphism with PAD (ABI) in subjects with type 2 diabetes.

## Research design and methods

### Study Sample

Patients in this study were participants in the Sandy Lake Complications Prevalence and Risk Factor Study, which was initiated to study the prevalence of, and risk factors for, complications of type 2 diabetes in aboriginal Canadians [29]. Sandy Lake, Ontario, is a remote Oji-Cree community, found at the 55^th ^parallel of latitude, in the subarctic boreal forest of central Canada. For this community-based, cross-sectional study, 189 eligible subjects with type 2 diabetes were enrolled, although the sample size varied for some variables given time-limited access to certain diagnostic equipment. Of these, 173 individuals had available DNA for *MTHFR *677C>T genotyping. For the determination of PAD, 140 subjects participated, with an overlap of 138 subjects who had both ABI and *MTHFR *677C>T genotype determination. Signed informed consent was obtained from all participants. The study was approved by both the Sandy Lake First Nation Band Council and the Mount Sinai Hospital Ethics Review Committee.

### Clinical characteristics and biochemical analysis

Measurements of fasting blood analytes, including glucose, lipid and lipoprotein concentrations, and percent glycoslyated hemoglobin (HbA_1c_), were performed as described [30]. Standardized procedures were used to measure blood pressure, height, weight, and waist circumferences [30]. Information on diabetes duration, diabetes treatment, and tobacco use, was obtained from interviewer-administered questionnaires [30]. Hypertensive individuals were defined as those subjects having either blood pressure exceeding systolic 130 mmHg and/or diastolic 80 mmHg, and/or receiving antihypertensive treatment.

### Diagnosis of peripheral arterial disease and intermittent claudication

The diagnosis of PAD was based on the ABI, which was measured using a blood pressure cuff and Doppler stethoscope. Systolic blood pressure was assessed at 3 sites on each side (brachial, posterior tibial, and dorsalis pedis arteries). Left and right ABI measurements were obtained by selecting the highest leg systolic blood pressure reading (either the posterior tibial systolic blood pressure or the dorsalis pedis systolic blood pressure) and dividing it by the mean brachial systolic blood pressure. The same individual performed all measurements. The use of ABI as a screening test for atherosclerotic disease has been previously validated [31], and test results have been shown to have high reproducibility when performed by trained professionals [32]. By convention, PAD was defined as an ABI of less than 0.95; subjects with an ABI greater than 1.40 were considered to have non-compressible vessels [29].

Intermittent claudication was assessed using the Rose (World Health Organization) questionnaire, with a positive score indicating the presence of leg pain, using the provided standard algorithm for diagnosis [33]. Leg pain was not considered intermittent claudication if it started when standing still or sitting, if it did not include the calves, or if it did not occur when walking up hill or hurrying [33].

### Genotyping *MTHFR *677C>T

An established procedure was used to genotype the *MTHFR *677C>T SNP [15]. Briefly, exon 4 was amplified using the following primers: 5'-CAA AGG CCA CCC CGA AGC and 5'-AGG ACG GTG CGG TGA GAG TG. Samples were amplified for 30 cycles, each of which consisted of denaturing at 94°C for 30 s, annealing at 58°C for 30 s, and extension at 72°C for 30 s. After *Hinf*I (New England Biolabs, Mississauga, Ontario, Canada) digestion of the resulting 245 bp fragment, the C allele yielded only a single 245 bp fragment, and the T allele yielded two fragments with sizes 176 and 69 bp. Electrophoresis in a 2.5% agarose gel followed by ethidium bromide staining and ultraviolet illumination allowed detection of the alleles.

### Statistical analysis

SAS version 8.2 (SAS Institute, Cary, NC) was used for all statistical comparisons. Data are presented as means ± standard deviation (s.d.) or as percentages for categorical variables. Logarithmic transformations (natural log) were used if data were not normally distributed. The transformed variables were used for parametric statistical analyses, but the untransformed values are presented in the tables. For continuous variables, differences between the groups were tested by the Student's *t *test; categorical variables were tested by χ^2 ^analysis or by Fisher's exact test. PAD was analyzed by multivariate logistic regression, with *MTHFR *677C>T genotype, duration of diabetes, current smoking habits, blood pressure, antihypertensive treatment, treatment for diabetes (insulin or oral agent), age, and sex included as independent variables. Deviation of genotype frequencies from those predicted by Hardy-Weinberg law was tested by χ^2 ^analysis. All statistical tests were two-sided and statistical significance was taken at nominal *P *< 0.05 for all comparisons.

## Results

### Characteristics of Oji-Cree type 2 diabetic patients

Study participants (35.5% male) had an average age of 46.7 ± 11.2 years for males and 45.8 ± 11.9 years for females, and a mean duration of diabetes of 8.27 ± 5.49 years for males and 8.90 ± 6.98 years for females. In comparison to males, females had significantly higher BMI (31.1 ± 5.2 *vs *29.0 ± 4.5 kg/m^2^, *P *= 0.020), and a lower frequency of hypertension (55.1% vs 77.6%, *P *= 0.0088). Approximately 14% had PAD, with PAD more frequent on the right side than the left (~12% *vs *~8%), and no significant difference between the numbers of males and females affected (14.3% males *vs *14.6% females, *P*=NS [0.96]). Slightly more than 1 out of 20 subjects had intermittent claudication (6.12% males *vs *62% females, *P *= NS [1.00]). Clinical attributes of the subjects, according to the presence or absence of PAD, are shown in Table 1. Significant differences between those with and without PAD were noted for total cholesterol concentrations and systolic blood pressure, with PAD-affected individuals having, on average, lower total cholesterol (*P *= 0.036) and higher systolic blood pressure (*P *= 0.0028). There were also fewer affected individuals taking antidiabetic drugs (*P *= 0.045).

**Table 1:** Characteristics of Oji-Cree type 2 diabetic patients according to the presence or absence of PAD (*max n *= *138*)

	**PAD present**		**PAD absent**		***P***
	**N**		**N**		
Sex (% male)	20	35.0	118	35.6	NS (0.96)
Age (years)	20	48.0 ± 11.2	118	45.8 ± 11.7	NS (0.44)
BMI (kg/m^2^)	20	30.4 ± 5.7	117	30.3 ± 4.9	NS (0.98)
Waist circumference (cm)	20	102 ± 13	117	104 ± 9	NS (0.52)
Current smokers (%)	20	65.0	118	47.5	NS (0.15)
Duration of diabetes (years)	20	8.70 ± 4.64	118	8.67 ± 6.76	NS (0.98)
Insulin treatment (%)	16	25.0	98	18.4	NS (0.51)
Intake of antidiabetic drugs (%)	16	31.3	98	58.2	0.045
Taking lipid medication (%)	20	5.0	118	16.1	NS (0.31)
Fasting glucose (mmol/L)	19	10.27 ± 2.79	111	9.96 ± 3.90	NS (0.48)
HbA_1c _(%)	20	8.30 ± 1.93	116	8.44 ± 2.29	NS (0.92)
Total cholesterol (mmol/L)	19	4.43 ± 0.63	114	4.93 ± 1.06	0.036
Plasma triglycerides (mmol/L)	19	1.54 ± 0.60	114	2.18 ± 2.68	NS (0.059)
Systolic BP (mmHg)	20	136 ± 21	118	123 ± 16	0.0028
Diastolic BP (mmHg)	20	74 ± 10	118	75 ± 9	NS (0.81)
Hypertensive (%)	20	70.0	118	61.9	NS (0.49)
CRP (mg/L)	19	6.86 ± 12.0	114	4.97 ± 5.87	NS (0.99)
ABI, left	20	0.96 ± 0.12	118	1.09 ± 0.08	<0.0001
ABI, right	20	0.93 ± 0.06	118	1.10 ± 0.08	<0.0001
Intermittent claudication (%)	20	15.0	118	4.2	NS (0.091)

Data are means ± s.d., unless otherwise indicated.

PAD, peripheral arterial disease: ABI <0.95; Hypertension: systolic BP ≥ 130 and/or diastolic BP ≥ 80 and/or antihypertensive treatment.

Abbreviations: BMI, body mass index; HbA_1c_, glycosylated hemoglobin; BP, blood pressure; CRP, C-reactive protein; ABI, ankle-brachial index; NS, not significant.

The overall allele frequencies for the 677C and 677T alleles were 0.89 and 0.11, respectively. All 677T carriers were heterozygotes (677C/T); no 677T/T homozygotes were found in the population sample studied. There was no significant difference found for the 677C>T genotype frequency between sex, with 16.3% of males and 24.7% of females, respectively, being carriers of the 677T allele (*P *= NS [0.25]). There was no significant deviation of the genotype frequencies, overall or according to sex, from those predicted by Hardy-Weinberg law.

Characteristics of subjects according to the presence or absence of the 677T allele are presented in Table 2. The thirty 677T carriers had significantly elevated BMI (32.6 ± 5.5 *vs *29.7 ± 4.7 kg/m^2^, *P *= 0.0050), and higher systolic blood pressure (132 ± 22 *vs *123 ± 16 mmHg, *P *= 0.021), in comparison to 677C/C homozygotes. There was no difference between 677T carriers and noncarriers regarding age, waist circumference, % current smoker, duration of diabetes, % hypertensive, diastolic blood pressure, mean ABI, and plasma concentrations of fasting blood glucose, HbA_1c_, total cholesterol, high-density lipoprotein cholesterol, low-density lipoprotein cholesterol, triglycerides, and C-reactive protein.

**Table 2:** Characteristics of Oji-Cree type 2 diabetic patients according to *MTHFR *677C>T genotype (*max n *= *138*)

	**N**	**677C/C**	**677C/T**	***P***
N (men/women)	138	108 (41/67)	30 (8/22)	NS (0.25)
Age (years)	138	46.4 ± 11.1	45.1 ± 13.5	NS (0.60)
BMI (kg/m^2^)	137	29.7 ± 4.7	32.6 ± 5.5	0.0050
Waist circumference (cm)	137	103 ± 9	106 ± 11	NS (0.063)
Current smoker (%)	138	50.0	50.0	NS (1.00)
Duration of diabetes (years)	138	8.53 ± 6.35	9.20 ± 7.01	NS (0.62)
Fasting glucose (mmol/L)	130	10.20 ± 3.91	9.28 ± 3.03	NS (0.33)
HbA_1c _(%)	136	8.36 ± 2.25	8.66 ± 2.18	NS (0.47)
Total cholesterol (mmol/L)	133	4.91 ± 1.07	4.67 ± 0.82	NS (0.27)
HDL-C (mmol/L)	133	1.21 ± 0.29	1.21 ± 0.29	NS (0.91)
LDL-C (mmol/L)	130	2.75 ± 0.71	2.70 ± 0.67	NS (0.78)
Plasma triglycerides (mmol/L)	133	2.20 ± 2.78	1.66 ± 0.60	NS (0.15)
Systolic BP (mmHg)	138	123 ± 16	132 ± 22	0.021
Diastolic BP (mmHg)	138	73.9 ± 8.9	77.4 ± 9.4	NS (0.077)
Hypertensive (%)	138	62.0	66.7	NS (0.64)
CRP (mg/L)	133	4.91 ± 6.65	6.53 ± 8.37	NS (0.19)
ABI, left	138	1.08 ± 0.09	1.05 ± 0.12	NS (0.26)
ABI, right	138	1.08 ± 0.09	1.07 ± 0.13	NS (0.73)

Data are means ± s.d., unless otherwise indicated.

Hypertension: systolic BP ≥ 130 and/or diastolic BP ≥ 80 and/or antihypertensive treatment

Abbreviations: BMI, body mass index; HbA_1c_, glycosylated hemoglobin; HDL-C, high-density lipoprotein cholesterol; LDL-C, low-density lipoprotein cholesterol; BP, blood pressure; CRP, C-reactive protein; ABI, ankle-brachial index; NS, not significant.

### Association of 677C>T SNP with peripheral arterial disease

Significantly more individuals carrying the 677T allele had PAD, as diagnosed by ABI (26.7% *vs *11.1%, *P *= 0.042); particularly when measured on the right side (23.3% *vs *8.33%, *P *= 0.047) (Table 3). However, no significant difference between carriers and non-carriers was noted for symptomatic intermittent claudication as determined by the Rose questionnaire [6.67% *vs *5.56%, *P *= NS (0.68)].

**Table 3:** PAD and intermittent claudication according to *MTHFR *677C>T genotype

	**677C/C n = 108**	**677C/T n = 30**	**OR (95% CI)**	P*
PAD, left or right (n, %)	12 (11.1%)	8 (26.7%)	2.91 (1.06, 7.97)	0.042
PAD, left (n, %)	7 (6.48%)	4 (13.3%)	2.22 (0.60, 8.16)	NS (0.25)
PAD, right (n, %)	9 (8.33%)	7 (23.3%)	3.35 (1.13, 9.93)	0.047

Intermittent claudication (n, %)	6 (5.56%)	2 (6.67%)	1.20 (0.26, 5.64)	NS (0.68)

* Fisher's exact test

Abbreviations: OR, odds ratio; PAD, peripheral arterial disease; NS, not significant.

Multivariate logistic regression (Table 4) revealed that *MTHFR *677C>T genotype was still significantly associated, albeit marginally, with increased risk of PAD [OR 3.54 (1.01, 12.4), *P *= 0.049], after adjustment for age, sex, duration of diabetes, hypertension, current smoking habits, and use of insulin or oral treatment for diabetes. None of these additional co-variables was significantly associated with PAD.

**Table 4:** Multivariate associations of risk factors with PAD

**Dependent variable**	**Independent variable**	**Unit**	**OR (95% CI)**	***P***
PAD, left or right	*MTHFR *677C>T	677C/T	3.54 (1.01, 12.4)	0.049
	oral treatment for diabetes	+	0.31 (0.082, 1.20)	NS (0.091)
	current smoker	+	3.04 (0.82, 11.2)	NS (0.095)
	hypertension	+	2.58 (0.58, 11.6)	NS (0.22)
	age	10 yr	1.36 (0.80, 2.29)	NS (0.26)
	duration of diabetes	5 yr	0.79 (0.45, 1.38)	NS (0.41)
	insulin administration	+	0.96 (0.18, 5.08)	NS (0.97)
	sex	male	1.00 (0.30, 3.34)	NS (0.99)

Hypertension: systolic blood pressure ≥ 130 and/or diastolic blood pressure ≥ 80 and/or antihypertensive treatment.

Abbreviations: OR, odds ratio; PAD, peripheral arterial disease; *MTHFR*, methylenetetrahydrofolate reductase; NS, not significant.

## Discussion

In Canadian Oji-Cree subjects with type 2 diabetes, we found that: 1) *MTHFR *677T carriers had an increased risk of PAD [OR 3.54 (1.01, 12.4), *P *= 0.049], adjusting for age, sex, duration of diabetes, hypertension, current smoking habits, and diabetes treatment; and 2) there was no significant association between *MTHFR *genotype and intermittent claudication, a much more advanced stage of PAD.

Our finding of a significant association between *MTHFR *genotype and PAD for subjects with type 2 diabetes is novel, to our knowledge. Only one other study to date has examined the relationship between PAD and *MTHFR *genotype in subjects with type 2 diabetes. In 135 patients with PAD and 219 controls, Ciccarone et al. found that though elevated homocysteine levels associated with the severity of PAD, as assessed by colour-duplex ultrasound, there was no significant association between *MTHFR *genotype and PAD [9]. Our observed association is of borderline statistical significance, yet given that under multivariate analysis *MTHFR *genotype was the most significant risk factor in comparison to other traditional risk factors, including smoking, hypertension, and duration of diabetes, investigation into its potential importance is warranted.

Though not significant, a 3-fold increased risk of PAD was observed for subjects who were current smokers. This finding stresses the importance of smoking cessation programmes in aboriginal communities, especially given the high prevalence of smoking in this population (~50% current smokers for both males and females). Taking preventative measures now is also critical considering the relatively young age of the population. Though the prevalence of PAD was ~14%, and the prevalence of intermittent claudication was only ~6%, the mean age of the study participants was under 50, younger than the majority of studies involving PAD [9,10,13]. A full expression of disease phenotypes associated with diabetes for the Oji-Cree is yet to be seen.

A limitation of our study was the small sample size. With less than 200 subjects included in each analysis, the power to detect significant associations was low. Most notably, the observation of no significant association for any of the well-known risk factors in the logistic regression analysis for PAD was likely due to the small sample size. In spite of the small sample size, the observation of an OR of greater than 3.5 for the *MTHFR *genotype and PAD suggests an association of notably strong magnitude which would be of interest to confirm in future studies.

Our results are also limited by the absence of both dietary information and plasma folate and homocysteine concentrations for our study subjects. Previous studies have found the prevalence of inadequate dietary folate intake in the Oji-Cree to be 37%, which is more than twice the average for the general Canadian population, and may contribute to compromised MTHFR enzyme activity [34]. These studies, however, took place in an era prior to the supplementation of the food source with folate, and thus may be an overestimation of the present situation. Further detailed clinical measurements would have helped to provide insight on the relationships between *MTHFR *genotype, plasma homocysteine concentration, and PAD. Other studies, however, have found significant associations for homocysteine levels with increased atherosclerosis, yet no significant association for *MTHFR *genotype [9,35], raising the question of possible alternative causes for hyperhomocysteinemia and questioning the real importance of the *MTHFR *genotype. Nonetheless, our data are consistent with the concept that *MTHFR *genotype may have a mechanistic role in the development and progression of atherosclerosis in the lower extremities.

## Conclusion

In conclusion, we have observed that the presence of the *MTHFR *677T allele was significantly associated with an ~3.5-fold increased risk for PAD, as assessed by ABI, in subjects with type 2 diabetes. The significance of the relationship between the *MTHFR *SNP and ABI was greater than that of any other common risk factor, including age, sex, duration of diabetes, hypertension, current smoking habits, or diabetes treatment. Since this variant is common, it could represent an important genetic determinant of PAD risk in the general population.

## Competing interests

The author(s) declare that they have no competing interests.

## Authors' contributions

RLP participated in the design of the study, genotyping, analysis of the data, and writing of the manuscript. MM participated in the on-site data collection and preformed all blood pressure measurements. AJGH, BZ, and SBH provided patients and data for the study, and assisted with manuscript revisions. RAH participated in the design of the study and writing of the manuscript. All authors read and approved the final manuscript.
